# Tablet PC Enabled Body Sensor System for Rural Telehealth Applications

**DOI:** 10.1155/2016/5747961

**Published:** 2016-01-18

**Authors:** Nitha V. Panicker, A. Sukesh Kumar

**Affiliations:** Department of ECE, College of Engineering, Trivandrum 695016, India

## Abstract

Telehealth systems benefit from the rapid growth of mobile communication technology for measuring physiological signals. Development and validation of a tablet PC enabled noninvasive body sensor system for rural telehealth application are discussed in this paper. This system includes real time continuous collection of physiological parameters (blood pressure, pulse rate, and temperature) and fall detection of a patient with the help of a body sensor unit and wireless transmission of the acquired information to a tablet PC handled by the medical staff in a Primary Health Center (PHC). Abnormal conditions are automatically identified and alert messages are given to the medical officer in real time. Clinical validation is performed in a real environment and found to be successful. Bland-Altman analysis is carried out to validate the wrist blood pressure sensor used. The system works well for all measurements.

## 1. Introduction

The population of elderly individuals is quickly expanding far and wide. The population of individuals of more than 65 years of age around the world will achieve 761 million by 2025, more than twofold of the 1990 figures [1]. In India, elderly adult is the fastest growing population, increasing from 6.7% in 1991 to 10% in 2021, who need more health care services [2]. The current telemedicine solutions focus more on the diagnosis and treatment of acute unwellness and expert consultation on emergency care especially for people in rural areas [3–6].

Hypertension and accidental falls are the two emerging public health problems and a barrier to active ageing [7]. Hypertension is defined as a systolic blood pressure (SBP) equal to or above 140 mm of Hg and/or diastolic blood pressure (DBP) equal to or above 90 mm of Hg. Normal levels of both systolic and diastolic blood pressure are particularly important for the efficient function of vital organs such as the heart, brain, and kidneys [8]. Hypertension is directly responsible for 57% of all stroke deaths and 24% of all coronary heart disease deaths in India [9, 10]. In an analysis of hypertension prevalence in India reveals that 33% of urban and 25% rural Indians are hypertensive [11]. The early detection and control of hypertension can reduce the risk of heart failure, heart attack, stroke, and kidney failure. The daily blood pressure monitoring leads to better blood pressure control. Self-monitoring of hypertension is recommended by [8] to manage the hypertension in patients across the world. The daily blood pressure monitoring removes white coat syndrome and thus leads to better blood pressure control.

Fall prevalence in India increases with age and is the highest in women and older adults [2]. All persons who experience a fall will remain on the ground or floor for more than 20 minutes prior to receiving assistance and this period of time spent immobile often affects their health. Getting timely help after a fall improves the chance of survival by 80% and increases the possibility of an independent living after fall. Therefore immediate detection of the fall is important to ensure that the person may receive timely assistance [12, 13].

Latest developments in the field of wireless technology can be utilized for monitoring. Wireless body area sensor network (WBASN) is a developing technology that interconnects tiny nodes with sensing capabilities in, on, or around a human body [14]. Recent trend in wearable health monitoring is to connect the biomedical sensors with mobile phones or tablet PC [15–19]. The use of mobile application for health care can provide great mobility and easy handling of information by a medical practitioner. Mobile phone/tablet PC enabled body sensor system is proposed in this work to measure hypertension, pulse rate, temperature, and accidental fall.

Table 1 provides a short review of wearable health monitoring systems for measuring physiological signals. Most of the studies focus on high risk cardiac and respiratory patients. Literature survey shows that very few efforts are taken to device a wireless health care solution which aims for easy handling of physiological data by a medical practitioner within a Community Health Center (CHC) or Primary Health Center (PHC) [20].

Community Health Centers (CHC) and Primary Health Centers (PHC) are managing a heavy patient load with the shortfall of doctors as well as supporting staffs [21]. So the developed system is validated in a Primary Health Center. Statistical analysis is carried out using Bland-Altman method. This work is the extension of the authors' previous research activity [22, 23].

This paper is organized as follows: Section 2 explores the monitoring systems.  Section 3 describes the materials and methods.  Section 4 explains the results followed by conclusions in Section 5.

## 2. Materials and Methods

### 2.1. Monitoring System Design

A wearable wrist sensor worn by the patient is used in this work for real time implementation. An embedded platform is used for acquiring the biological information from the wrist sensor and transmitting to a mobile phone/tablet PC running on android platform which is monitored by a medical staff.

The hypertension status of the person under test is immediately sent to the tablet PC and automatic alert facility for hypertensive patients is sent to the medical officer through short message service (SMS) facility. The proposed telehealth system architecture with remote monitoring facility is shown in Figure 2.

**Figure 1:**
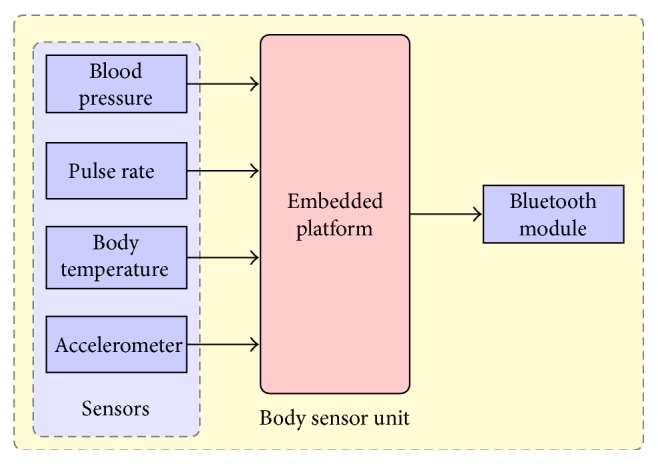
Block diagram of the developed body sensor unit.

**Figure 2:**
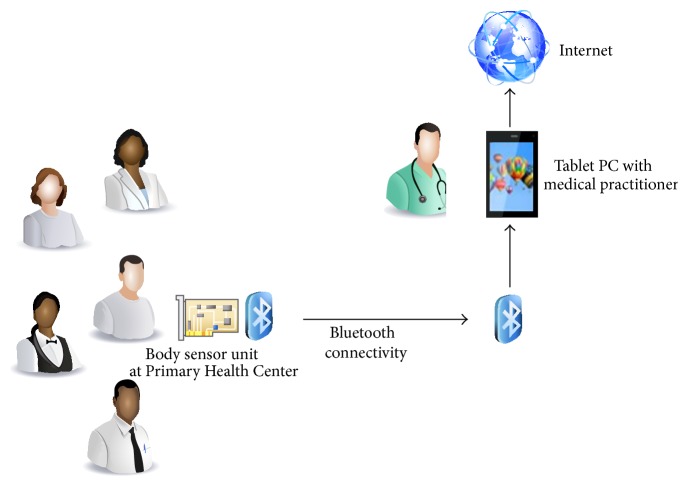
Proposed telehealth system architecture with remote monitoring facility.

The block schematic of the body sensor unit developed for this work is shown in Figure 1. The developed system consists of Body sensor unit and a mobile phone/tablet PC with Android Operating System. Body sensor unit consists of embedded platform, blood pressure sensor, pulse rate sensor, temperature sensor, accelerometer sensor, and bluetooth module.

#### 2.1.1. Sensors

The body sensor unit, connected to the patient at the health center, consists of the following sensor units.

*(a) Blood Pressure/Pulse Rate Sensor*. Arterial pressure is defined as the hydrostatic pressure exerted by the blood over the arteries as a result of the heart left ventricle contraction [24]. Blood pressure is represented by two numbers: systolic blood pressure (SBP) and diastolic blood pressure (DBP). SBP is the highest blood pressure reached by the arteries during ventricular contraction and DBP is the lowest blood pressure reached during ventricular relaxation. Normal adult pressure is an SBP of 120 mm of Hg and a DBP of 80 mm of Hg.

A noninvasive blood pressure (NIBP) sensor working on oscillatory principle is connected at the wrist of the subject. Wrist sensor measures systolic blood pressure (SBP), diastolic blood pressure (DBP), and pulse rate (PR) of the subject under test. The proper cuff and bladder size used in the assessment of blood pressure is important for accurate measurement. The use of a cuff that is too short and narrow for a given arm results in erroneously high blood pressure measurement. Use of a cuff that is too large results in erroneously low blood pressure measures. Wrist blood pressure sensor used in this work has a cuff with a bladder that is 120 mm wide and 250 mm long.

*(b) Temperature Sensor*. The LM35 series are precision integrated-circuit temperature sensors, whose output voltage is linearly proportional to the Celsius (Centigrade) temperature [25]. The LM35D is rated to operate over a 0 degree to +100 degree C temperature range. For body temperature measurement the package sensor should touch the wrist of the subject. The metal case LM35 works better to provide stable and correct output.

*(c) Accelerometer Sensor*. A wearable sensor system called Smart Wristlet, providing 24-hour fall detection service, is discussed in [26]. The above system collects data that reflect the wearers activity from multiple channels. MMA 7361L Accelerometer is used in the current work for fall detection [27]. MMA 7361L is an MEMS accelerometer which has 3-axis acceleration signals and one 0 g detect feature. Algorithm for fall detection is based on thresholding technique. When the *x*-, *y*-, and *z*-axes are experiencing free fall, 0 g pin (6th pin) will generate a high voltage which can be used to detect person fall. This signal is given to the interrupt pin of microcontroller in order to give highest priority to this event.

#### 2.1.2. Embedded System

Embedded technology is implemented to perform a specified task. The programming of PIC 16f877A microcontroller is done using embedded C [28]. This is a high-performance 40-pin microcontroller. It has 8 kilo bytes program memory, 368 bytes of flash programmable memory, and 256 bytes of EEPROM. PIC is an extremely efficient microcontroller which runs with typically less program memory than its competitors. MPLAB IDE is used for writing, compiling, and uploading the program into the PIC microcontroller development board.

#### 2.1.3. Bluetooth Module

Bluetooth wireless communication has the advantage of higher data rate and easy interfacing with personal digital assistant such as a smart phone or a tablet PC. JYMCU HC-05 Bluetooth Module is used in this work which connects to the embedded platform for wireless transmission. Bluetooth module is connected at the software serial port of the microcontroller.

#### 2.1.4. Tablet PC with Android Operating System

Android Studio IDE is used to develop the application for tablets/smart phones with Android Operating System [29]. Android is a Linux-based Operating System for mobile devices such as smartphones and tablet computers. Proper device drivers must be installed for connecting and loading applications into the system which have Android Operating System [30].

A low cost high performance tablet PC (Digiflip XT 811) with Kitkat 4.4 operating system is used in this work for real time implementation. Tablet PC has the advantage of large screen size and better functionality compared to a mobile phone. The android application receives the data transmitted from the body sensor unit via bluetooth communication facility. An android application developed for tablet PC is used for the purpose of real time acquisition of data, comparison with threshold value, storage in SQL database, and alert mechanism generation using SMS facility.

The android application initiates GPS services provided by the mobile phone network. While executing the subroutine for fall alert the developed android application collects location information via services given by the network provider or GPS provider. Information such as latitude, longitude, and exact address are collected by proper subroutines. This information is sent along with the text of fall alert message.

### 2.2. Validation of Wrist Sensor

#### 2.2.1. Study Population

The examinations have been carried out in Rural Primary Health Center in Andoorkonam, Trivandrum district of Kerala, India. The measurements were authorized by the Office of the District Medical Officer. Patients having Blood pressure variations or under medication for hypertension were directed by the medical officer for measurement. Overall 291 patients including healthy staffs in the PHC have been included.

#### 2.2.2. Method of Investigation

The aim of the validation of the device is to understand the reliability of the data obtained using the wrist sensor. The study also aims to get the feedback of the patients under test about the wrist sensor for blood pressure measurement. The study was conducted at a rural Primary Health Center in Trivandrum and the procedures in this study were approved by the District Medical Officer (DMO) and the medical officer, Andoorkkonam. The study was conducted in accordance with guidelines provided by the medical officer and under the supervision of two trained staff nurses. All subjects were informed and got consent for this study.

**Figure 3:**
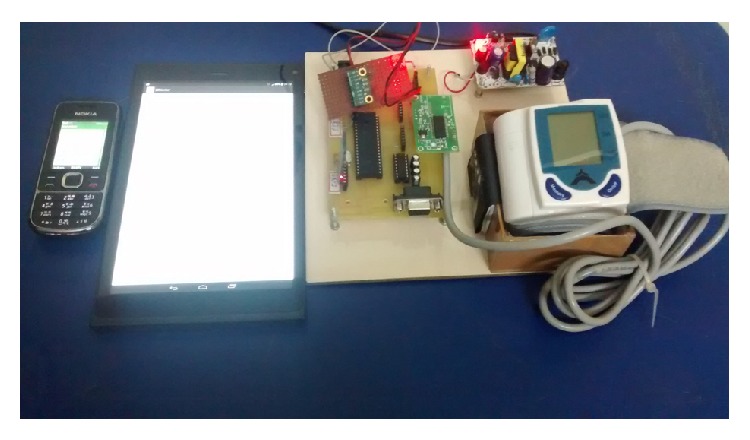
Hardware setup (BSU, microcontroller development board, Tablet PC, and mobile phone).

**Figure 4:**
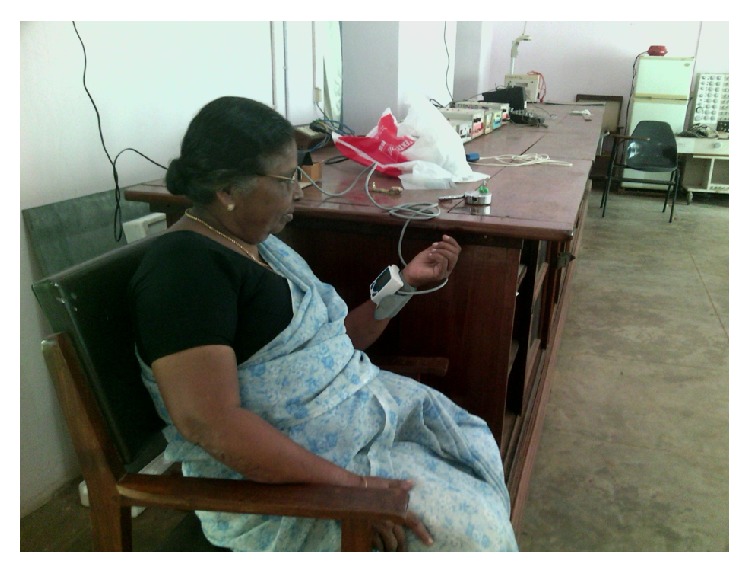
Conduction of testing with wrist device.

291 subjects suspected to have hypertension/under medication for hypertension were suggested for BP measurement by the medical officer. 113 male subjects and 178 female subjects were first measured by the sphygmomanometric method and then by the digital wrist sensor based on oscillometric method. Hardware setup (BSU, microcontroller development board, tablet PC, and mobile phone) is shown in Figure 3. The conduction of testing is shown in Figure 4. The sensor values taken by the oscillometric method were stored in the SQL database of the mobile phone/tablet PC.

**Figure 5:**
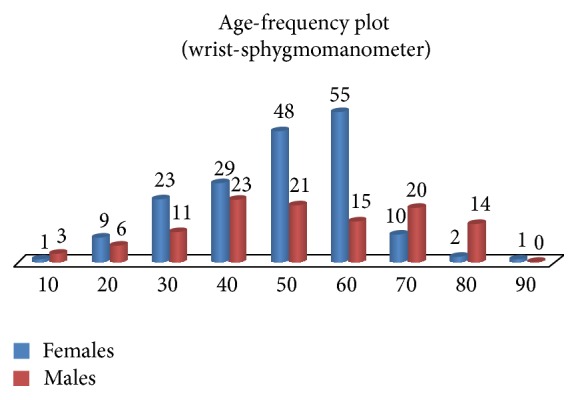
Age and frequency of subjects under study.

**Figure 6:**
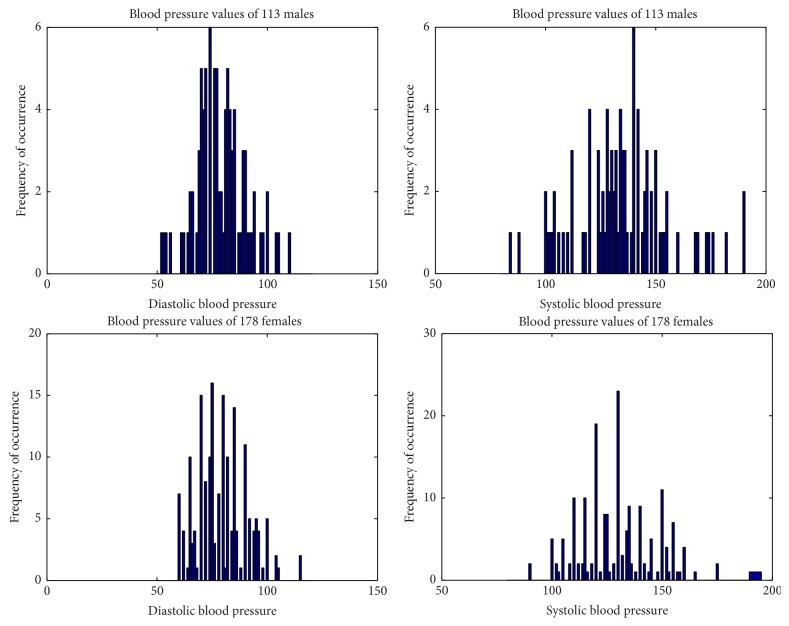
Frequency of SBP and DBP for male and female subjects using sphygmomanometric method.

Age frequency plot of males and females in different age groups under study are shown in Figure 5. This method of measuring was compared against sphygmomanometric method (auscultatory) which is considered as the gold standard of blood pressure measurement. First the subjects have undergone sphygmomanometric method with cuff placed at the arm. The frequency of occurrence of SBPs and DBPs using sphygmomanometric method for male and female subjects is shown in Figure 6.

The same subject was asked to take measurements using the wrist sensor also. The sensor was suitably positioned at the heart level in order to eliminate errors caused by position artifacts. The pair of data were used for validation of the wrist sensor. The statistical analysis was done for systolic blood pressure and diastolic blood pressure using Bland-Altman method.

## 3. Results

### 3.1. Experimental Results

The system was tested in a practical environment and found to be most successful. The information was transmitted successfully to the tablet PC and stored in the SQL database by the developed android application. Patients with hypertension (SBP equal to or above 140 mm of Hg and/or DBP equal to or above 90 mm of Hg) were correctly identified and automatic alert messages were generated and sent to the medical officer by the developed android application. Low blood pressure conditions (SBP below 90 mm of Hg and DBP mm of Hg below 60) were also identified. Normal and high body temperature were identified and transmitted to the tablet PC via bluetooth module.

**Figure 7:**
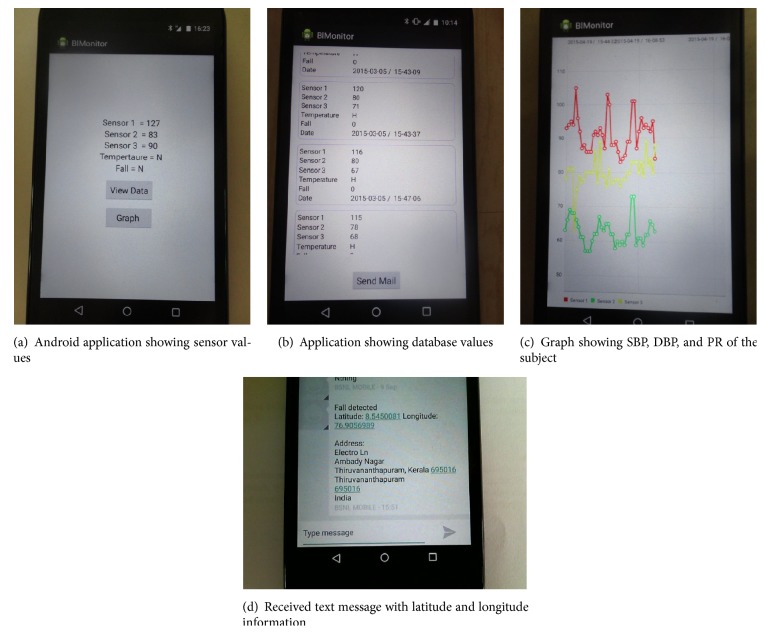
Screen shots of output observed on the tablet PC.

Screen shots of outputs obtained are shown in Figure 7. Sensor 1 indicates SBP, sensor 2 gives DBP, and sensor 3 gives pulse rate of the subject.  Figure 7(d) shows the automatic alert message received by the medical officer. This facility can be applied for elderly care in out-of-hospital or home environment. The latitude and longitude information can be used to identify the exact location of the patient so that he/she can be precisely located.

### 3.2. Statistical Analysis

The validations were carried out largely according to the recommendations of the Association for the Advancement of Medical Instrumentation (AAMI). AAMI recommends the inclusion of a larger patient number and a greater number of subjects in an older age group in order to test the monitors' performances over a wider range of patient characteristics. The criteria require device to observe discrepancy within 5 ± (8 s.d.) mm of Hg for mean difference and standard deviation.

**Figure 8:**
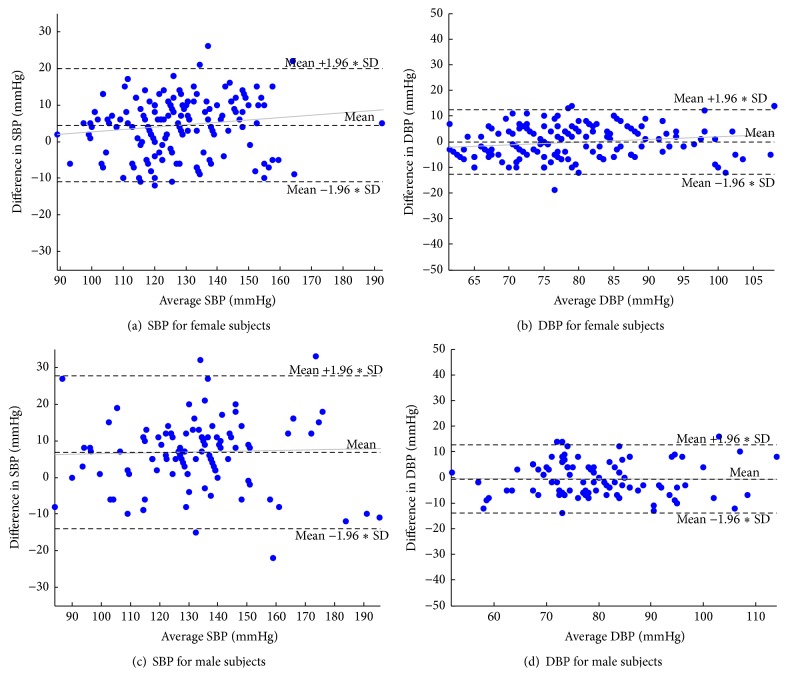
Scatter plot of difference and average of parameters taken by wrist sensor method and sphygmomanometric method.

**Figure 9:**
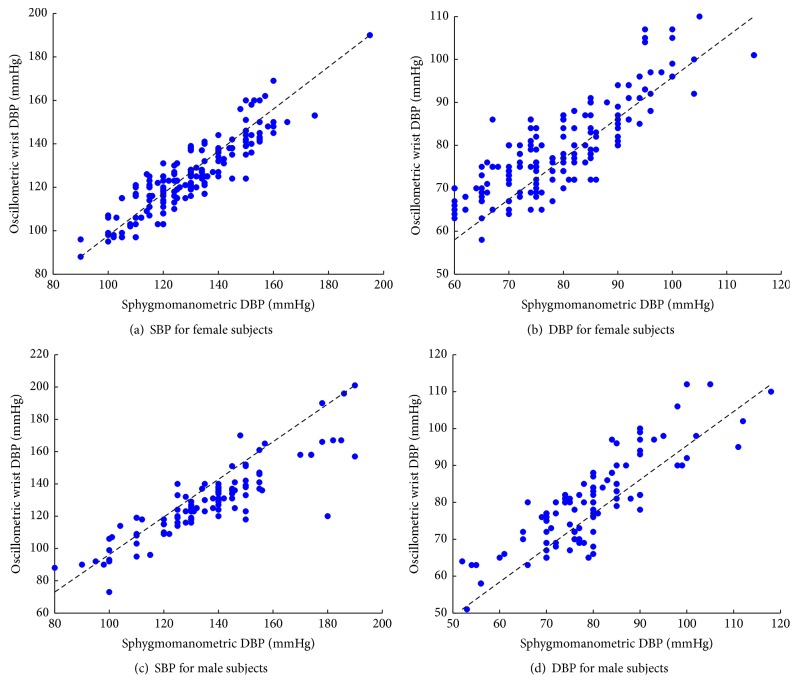
Scatter plot of of wrist sensor on that of sphygmomanometric method.

Bland-Altman analysis was carried out to show the agreement between the two tests. The scatter plot of difference and average of SBP and DBP taken by wrist sensor method and sphygmomanometric method of female population and male population are shown in Figure 8. A comparison is also made by plotting wrist sensor values on that of sphygmomanometric method which is shown in Figure 9. The results show very good agreement between the two methods.  Table 2 provides the bias ± standard deviation of the comparison result. The device passed the AAMI criteria except for SBP of male subjects. Thus wrist sensor under proper supervision can be used instead of sphygmomanometric method for hypertension monitoring.

The accuracy of wrist sensor is calculated by identifying true positives, false positives, true negatives, and false negatives. The obtained accuracy is shown in Table 3. The accuracy of fall alarm is calculated with 20 simulated falls of patients aged 20–40 including 12 males and 8 females. The obtained accuracy is shown in Table 4.

## 4. Discussion

The aim of this comparison was the analysis for clinical suitability of the wrist blood pressure sensor for family and community health care application. A comparison was done with sphygmomanometric method, which served as a reference device because of its wide distribution and acceptance. The parameters under investigation were the systolic and diastolic blood pressure.

The trials showed satisfactory acceptance from the patient side because of the low discomfort caused by the wrist blood pressure sensor. Bland-Altman analysis shows very good agreement between the two methods of measurement.

## 5. Conclusion

Wrist blood pressure sensor is more suitable in subjects with obesity having large arm sizes. Wrist blood pressure sensor does not require patients to remove clothing while taking measurements and it provides less discomfort compared to arm cuff monitors during inflation.

The measurement taken at any time is successfully transmitted to the tablet PC and is stored in the SQL database of the android phone/tablet by the developed application without any manual intervention. This enables the medical officer to ascertain whether a drug is effective or not. The developed system is less expensive and causes less inconvenience for the patients.

The validation conducted for the oscillometric wrist technique yields good results which shows good correlation between the BP readings taken with a sphygmomanometer and oscillometric wrist sensor. Patient's opinion about wrist sensor was promising. The device passed the AAMI criteria for SBP and DBP measurement except for male systole data. This study suggests that wrist devices can produce reliable and accurate results. The principal easiness of wrist blood pressure sensor based on oscillometric methods offers the opportunity for wide spread use. This may ultimately lead to an improvement in common efforts to prevent hypertension.

A modern approach of health monitoring and management is put into practice by the experiment conducted using the developed system. The ever-increasing workload of physicians can be significantly reduced by the use of such systems.

## Figures and Tables

**Table 1:** Review on wearable systems for healthcare.

Project title	Hardware description	Medical application
AMON [31], 2004	Wrist worn device, GSM	High risk cardiac/respiratory patients
LifeGuard [32], 2005	Microcontroller, serial cables	Monitoring in extreme environment (terrestrial application)
RTWPMS [33], 2006	Cordless phone, RS232 cables	General remote health monitoring
SmartVest [34], 2008	Woven sensors	General remote health monitoring
Wearable Belt [35], 2010	Chest worn device, serial cables	General monitoring of cardiac and respiratory patients
ZigBee-Based Monitoring [36], 2012	Wrist worn device, ZigBee Technology	General remote sensing of heart rate and temperature
Smart-Clothes Platform [37], 2013	Woven sensors, USB cables	General monitoring of cardiac and respiratory patients

**Table 2:** Correlation study and Bland-Altman analysis results for device comparison.

Subject	Parameter	Cab	Bias ± SD	Limits of agreement
Male	Systole	0.8804	6.9027 ± 10.6544	1.0023
Male	Diastole	0.8386	−0.6637 ± 6.7818	0.6380
Female	Systole	0.8920	4.4775 ± 7.9157	0.5933
Female	Diastole	0.8284	−0.1798 ± 6.4160	0.4809

**Table 3:** Sensitivity, specificity, and accuracy when comparing sphygmomanometer and wrist method.

Subject	Sensitivity	Specificity	Accuracy
Male (113)	52.63%	94.64%	73.45%
Female (178)	63.49%	95.65%	84.27%
Total (291)	58.33%	95.32%	80.07%

**Table 4:** Sensitivity, specificity, and accuracy for fall detection system.

Type of fall	Sensitivity	Specificity	Accuracy
Hard fall (stand position towards floor)	87.5%	75%	80%
Hard fall (stand position towards bed)	71.42%	69.23%	70%
Soft fall (stand position towards floor)	62.5%	58.33%	60%
Soft fall (stand position towards bed)	55.55%	54.54%	55%
